# Demographic and Clinical Factors Associated With Diagnostic Confidence in Interstitial Lung Disease: Findings From the Pulmonary Fibrosis Foundation Patient Registry

**DOI:** 10.1016/j.chpulm.2024.100084

**Published:** 2024-07-15

**Authors:** Mary Beth Scholand, Sachin Gupta, Kevin R. Flaherty, Rosalinda V. Ignacio, Zhongze Li, Ayodeji Adegunsoye

**Affiliations:** a Department of Internal Medicine, University of Utah, Salt Lake City, UT; b Genentech Inc, South San Francisco, CA; c Division of Pulmonary and Critical Care Medicine, Department of Internal Medicine, University of Michigan Ann Arbor, MI, USA; d Department of Biostatistics, University of Michigan Ann Arbor, Ann Arbor, MI; e Section of Pulmonary and Critical Care Medicine, University of Chicago, Chicago, IL

**Keywords:** diagnostic confidence, idiopathic pulmonary fibrosis, interstitial lung disease, Pulmonary Fibrosis Foundation Patient Registry

## Abstract

**Background:**

Accurate diagnosis of interstitial lung disease (ILD) can be challenging. Accordingly, clinicians may attribute a diagnostic certainty based on guideline criteria and clinical judgment. However, further research is needed to refine this approach and improve diagnostic clarity.

**Research Question:**

What are the real-world factors associated with diagnostic confidence in fibrotic ILD?

**Study Design and Methods:**

Data were included from all patients enrolled in the Pulmonary Fibrosis Foundation Patient Registry from March 2016 to August 2018. Baseline demographic and clinical characteristics were collected at enrollment, or at the test date closest to the date of consent for longitudinal measures. Descriptive analyses were performed separately for all participants, and for subgroup participants with idiopathic pulmonary fibrosis (IPF) and participants with non-IPF ILD, stratified by the level of investigator diagnostic confidence (high vs medium/low) assigned at registry enrollment. Adjusted ORs and 95% CIs were calculated using multivariable logistic regression, with the aforementioned characteristics as predictors.

**Results:**

Data up to April 2022 from 1,992 participants were included. In adjusted logistic regression analyses among all participants, antifibrotic use (OR, 1.51; 95% CI, 1.09-2.07), longer time since diagnosis (OR, 0.94; 95% CI, 0.89-0.98) at the research unit of 365 days, and diabetes (OR, 2.56; 95% CI, 1.01-6.44) were significantly associated with higher diagnostic confidence, and non-IPF idiopathic interstitial pneumonia (vs IPF; OR, 0.36; 95% CI, 0.24-0.55), insurance - other (OR, 0.65; 95% CI, 0.43-0.97), and Hispanic ethnicity (OR, 0.54; 95% CI, 0.31-0.94) were significantly associated with lower diagnostic confidence. Factors associated with diagnostic confidence in the IPF and/or non-IPF ILD groups included age, male sex, region, immunomodulatory medication use, multidisciplinary team discussion, surgical lung biopsy, and definite high-resolution CT pattern.

**Interpretation:**

These findings suggest that certain demographic and clinical factors may influence physicians’ confidence in diagnosis of IPF and non-IPF ILD. Tailored physician education may help to reduce biases and improve consistency in diagnosis.

**Take-home Points**

**Study Question:** What are the real-world factors associated with diagnostic confidence in fibrotic interstitial lung disease (ILD)?

**Results:** Our results suggest that clinicians were more confident in the diagnosis of idiopathic pulmonary fibrosis (IPF) than non-IPF ILD, and that many factors including multidisciplinary discussion, type of treatment, and Hispanic ethnicity may be associated with diagnostic confidence in patients with IPF and/or patients with non-IPF ILD.

**Interpretation:** Multiple demographic and clinical factors may influence physicians’ confidence in the diagnosis of IPF and non-IPF ILD.

Accurate diagnosis of fibrotic interstitial lung disease (ILD) is important because it informs clinicians of prognosis and management options.^1^ However, even with clinical experience and the availability of diagnostic guidelines,^2^ differentiating between specific ILDs is challenging, and diagnosis for some patients may be unclear or lack confidence.^1^^,^^3^ Moreover, there is a lack of objective guidance on incorporation of patient and clinical variables (eg, age, disease behavior), which may influence diagnostic confidence.^2^, ^3^, ^4^, ^5^ In the absence of a definite diagnosis, one approach to the evaluation of patients with suspected idiopathic pulmonary fibrosis (IPF) is to assign a working diagnosis that can be reviewed and updated as more information becomes available.^6^ Alternatively, physicians may allocate a level of confidence to a diagnosis of fibrotic ILD, with patients assigned a confident or provisional diagnosis, or categorized as having unclassifiable ILD, based on guideline criteria and clinical judgment.^3^ However, both of these approaches incorporate clinician opinion, and further research is needed to improve diagnostic clarity.^3^^,^^6^

To better understand real-world factors associated with diagnostic confidence in fibrotic ILD, we explored the demographic and clinical characteristics of patients with IPF and non-IPF ILD enrolled in the Pulmonary Fibrosis Foundation (PFF) Patient Registry (referred to as the registry from this point on), stratified by the level of diagnostic confidence at enrollment.

## Study Design and Methods

### Study Population

The registry is an observational database that collects longitudinal demographic and clinical data from patients with IPF and non-IPF ILDs across the United States. In line with the target enrollment, around 60% of patients enrolled in the registry had a diagnosis of IPF made or confirmed at a PFF center.^7^ Patients with non-IPF ILDs included in the registry were diagnosed with idiopathic interstitial pneumonias, collagen vascular diseases, or hypersensitive pneumonitis; more information on the types of non-IPF ILDs included in the registry can be found in e-Table 1.^7^ Adult patients with a diagnosis of ILD who had not undergone lung transplantation, had received care at one of the registry centers, and had provided informed consent were eligible for inclusion in the registry. Enrollment started in March 2016 and completed in August 2018.^7^, ^8^, ^9^

This analysis included data collected from March 2016 to April 2022 from all patients enrolled in the registry. Participants were stratified by the level of investigator confidence in diagnosis (high, medium, or low) assigned at the time of entry into the registry.

The registry was approved by the institutional review board at each participating site, and all patients provided written informed consent. This analysis used deidentified patient data and was considered exempt from institutional review board review.

### Outcomes

Patient characteristics described in this study were previously recorded in the registry database^7^ and were included in this analysis due to their relevance to the biological mechanism of ILD. Baseline characteristics, including demographics and clinical characteristics, were collected at the time of enrollment. Age was calculated as the number of complete years from the participant’s date of birth to the date of informed consent. Participants were classified as living in an urban or rural area by US Census classification; rural (micropolitan and noncore regions) or urban (large, medium, and small metropolitan regions) classification was based on mapping of participants’ reported Zip codes to county Federal Information Processing Standard codes. Region (West, Midwest, South, and Northeast) was based on the state where the site is located, as in a previous registry analysis.^7^ Clinical characteristics included comorbid conditions, history of biopsy, lung function, medication use, definite usual interstitial pneumonia (UIP) pattern on high-resolution CT (HRCT) scan (patients with IPF only [yes/no]), and patient-reported outcomes (PROs) (including the Short-Form 6-Dimension tool, Leicester Cough Questionnaire, University of California, San Diego Shortness of Breath Questionnaire, and Fatigue Severity Scale).

Participants were classified as having used antifibrotics, immunomodulatory medications, proton pump inhibitors, supplemental oxygen, or pulmonary rehabilitation if the date of consent was within the start and end dates of medication use. For longitudinal measures (eg, % predicted FVC, % predicted carbon monoxide diffusing capacity [Dlco]) uncorrected, the value with a test date closest to the date of consent was used.

### Statistical Analysis

Descriptive analyses were performed separately for all participants, participants with IPF, and participants with non-IPF ILD, stratified by diagnostic confidence level. Baseline demographic and clinical characteristics were compared between the confidence levels using χ^2^ test or analysis of variance.

Due to the small sample sizes of the medium and low confidence groups, the study team elected to combine both categories into one; thus, diagnostic confidence was revised to a binary response (high vs medium/low). Logistic regression analyses were performed to estimate the relationship between confidence level (high vs medium/low) and baseline characteristics.

Unadjusted ORs and 95% CIs were calculated from separate simple logistic regression models, with each characteristic as a predictor. Adjusted ORs and 95% CIs were calculated from multivariable logistic regression models with all characteristics as predictors. For all analyses, *P* < .05 was considered statistically significant. ORs and CIs were calculated for continuous characteristics expressed in units as follows: 10 years for age, 365 days for the days from consent to diagnosis, and 40.2 km for distance to study site. To note, since days from consent to diagnosis have negative values by definition (ie, date of diagnosis is prior to date of enrollment to the registry because ILD diagnosis is an eligibility criterion), an OR < 1 implies lower odds (ie, lower probability) for high diagnostic confidence for shorter time interval. Equivalently, longer time interval implies higher odds (ie, higher probability) for higher diagnostic confidence.

## Results

### Descriptive Analysis of Baseline Characteristics Stratified by Diagnostic Confidence Level

In total, 1,992 participants were enrolled in the registry, including 1,227 participants (61.6%) with IPF and 765 participants (38.4%) with non-IPF ILD. The mean age ± SD was 67.7 ± 10.2 years, 1,251 participants (62.8%) were male, and 1,776 participants (89.2%) were White. Mean % predicted FVC ± SD was 68.0% ± 18.2%, and mean uncorrected Dlco ± SD was 42.4% ± 17.7%. In total, 902 participants (45.3%) required supplemental oxygen, and 794 (39.9%) were using antifibrotics. Chronic comorbidities included sleep apnea (n = 503, 25.3%), coronary artery disease (n = 418, 21.0%), diabetes (n = 378, 19.0%), and pulmonary arterial hypertension (PAH) (n = 153, 7.7%).

Among all participants, a history of surgical lung biopsy (SLBx), ILD subtype (particularly IPF diagnosis), antifibrotic use, and longer time from consent to diagnosis were significantly associated with higher diagnostic confidence (all *P* < .001) (Table 1). Additional baseline characteristics that were significantly associated with diagnostic confidence (all *P* < .05) included race (White, Black or African American, and other racial groups), lung function (categorical % predicted FVC), and various comorbidities (COPD, pulmonary vascular disease, PAH) (Table 1).

**Table 1:** Baseline Demographic and Clinical Characteristics at Enrollment by Diagnosis Confidence Level (High vs Medium/Low): All Participants

Characteristic	High Confidence (n = 1,520)	Medium/Low Confidence (n = 472)
Age, y	67.7 ± 10.4 (69.0)	67.8 ± 9.7 (69.0)
Male	954 (62.8)	297 (62.9)
**Race**^a^^**,**^^b^		
White	1,354 (89.1)	422 (89.4)
Black or African American	88 (5.8)	18 (3.8)
Other	35 (2.3)	20 (4.2)
Hispanic	86 (5.9)	38 (8.4)
Rural	190 (12.5)	54 (11.4)
Region		
West	244 (16.1)	89 (18.9)
Midwest	353 (23.2)	101 (21.4)
South	579 (38.1)	182 (38.6)
Northeast	344 (22.6)	100 (21.2)
Insurance - private	1,499 (98.6)	463 (98.1)
Insurance - Medicare	929 (61.1)	268 (56.8)
**Insurance - others**^b^	159 (10.5)	66 (14.0)
Smoking history	877 (57.7)	285 (60.4)
No. of other chronic conditions	1.5 ± 1.4 (1.0)	1.6 ± 1.4 (1.0)
Other chronic conditions		
GERD	253 (16.6)	64 (13.6)
CAD	318 (20.9)	100 (21.2)
CHF	62 (4.1)	22 (4.7)
**COPD**^b^	122 (8.0)	55 (11.7)
Diabetes	301 (19.8)	77 (16.3)
**PAH**^b^	127 (8.4)	26 (5.5)
**PVD**^b^	23 (1.5)	15 (3.2)
Family history of ILD	226 (14.9)	57 (12.1)
**Biopsy**^c^	583 (38.4)	145 (30.7)
Transbronchial biopsy	93 (6.1)	35 (7.4)
**SLBx**^d^	496 (32.6)	101 (21.4)
FVC % predicted, %	68.0 ± 17.8 (67.1)	67.9 ± 19.6 (66.2)
**FVC % predicted, categorical**^b^		
< 50%	224 (14.7)	87 (18.4)
50% to < 80%	882 (58.0)	238 (50.4)
≥ 80%	358 (23.6)	121 (25.6)
Dlco uncorrected % predicted, %	42.1 ± 17.7 (40.2)	43.5 ± 17.7 (40.4)
Dlco uncorrected % predicted, categorical		
< 50%	1,024 (67.4)	292 (61.9)
50% to < 80%	340 (22.4)	113 (23.9)
≥ 80%	41 (2.7)	18 (3.8)
Supplemental oxygen use	697 (45.9)	205 (43.4)
**ILD subtype**^d^		
IIP-IPF	977 (64.3)	250 (53.0)
IIP-non-IPF	122 (8.0)	94 (19.9)
Collagen vascular disease/autoimmune disease	271 (17.8)	62 (13.1)
Hypersensitivity pneumonitis	108 (7.1)	48 (10.2)
Other forms of ILD	42 (2.8)	18 (3.8)
MDT discussion	627 (41.3)	197 (41.7)
**Antifibrotic medications (pirfenidone or nintedanib)**^d^	657 (43.2)	137 (29.0)
Immunomodulatory medications	279 (18.4)	87 (18.4)
Proton pump inhibitor	261 (17.2)	78 (16.5)
**Days from consent to diagnosis**^d^	−1,064.1 ± 1,138.1 (−722.8)	−833.8 ± 984.3 (−502.0)

Data are No. (%) or mean ± SD (median) unless otherwise indicated. Categories with *P* < .05 are highlighted in boldface font. CAD = coronary artery disease; CHF = congestive heart failure; Dlco = carbon monoxide diffusing capacity; GERD = gastroesophageal reflux disease; IIP = idiopathic interstitial pneumonia; ILD = interstitial lung disease; IPF = idiopathic pulmonary fibrosis; MDT = multidisciplinary team; PAH = pulmonary arterial hypertension; PVD = pulmonary vascular disease; SLBx = surgical lung biopsy.

a Excludes 55 participants with race not reported or unknown race.

b *P* < .05.

c *P* < .01.

d *P* < .001.

In participants with IPF, biopsy (including SLBx), antifibrotic use, a definite UIP pattern on HRCT scan, longer time since diagnosis, history of multidisciplinary team (MDT) discussion, and residence in a rural area were all associated with greater diagnostic confidence (all *P* < .05) (e-Table 2). In contrast, obesity, immunomodulatory medications, pulmonary vascular disease, and insurance were associated with lower confidence. In participants with non-IPF ILD, longer time since diagnosis, presence of PAH, immunomodulatory medications, and SLBx were associated with greater diagnostic confidence, whereas male sex, increasing age, and a history of MDT discussion were associated with lower confidence in diagnosis (all *P* < .05) (e-Table 3). Race was significantly associated with diagnostic confidence for both IPF and non-IPF participants, whereas lung physiology and PROs were not associated with diagnostic confidence.

### Logistic Regression Analyses

The results from the unadjusted logistic regression analyses are presented in e-Table 4. Table 2 and e-Table 5 show the results of the adjusted logistic regression analyses to estimate the relationship of diagnostic confidence and baseline characteristics controlling for all other covariates in the model. Based on the adjusted logistic regression analyses, among all participants, antifibrotic use, diabetes, and longer time from consent to diagnosis were significantly associated with higher diagnostic confidence, whereas ILD subtype of non-IPF idiopathic interstitial pneumonia, Hispanic ethnicity, and insurance - others were significantly associated with lower diagnostic confidence (Table 2).

**Table 2:** Adjusted Logistic Regression Analyses for Diagnosis Confidence Level (High vs Medium/Low)

Variables^a^	All Participants^b^		Participants With IPF^c^		Participants With Non-IPF ILD^d^	
	Adjusted OR (95% CI)^e^^,^^f^	*P* Value	Adjusted OR (95% CI)^e^^,^^f^	*P* Value	Adjusted OR (95% CI)^e^^,^^f^	*P* Value
Age^g^	0.93 (0.79-1.09)	.386	1.12 (0.86-1.45)	.406	0.76 (0.6-0.95)	**.018**
Male	0.89 (0.66-1.2)	.440	1.34 (0.87-2.06)	.180	0.57 (0.37-0.88)	**.011**
Race						
White (reference)	…	…	…	…	…	…
Black or African American	1.25 (0.64-2.44)	.506	0.82 (0.19-3.5)	.794	1.25 (0.57-2.72)	.577
Other	0.53 (0.25-1.12)	.097	0.71 (0.23-2.16)	.542	0.52 (0.16-1.7)	.277
Hispanic	0.54 (0.31-0.94)	**.031**	0.32 (0.13-0.77)	**.011**	0.86 (0.4-1.88)	.711
BMI	1.01 (0.98-1.04)	.556	1.02 (0.98-1.07)	.266	0.98 (0.94-1.02)	.269
Rural	1.17 (0.79-1.71)	.432	1.4 (0.8-2.45)	.245	0.98 (0.55-1.75)	.941
Region						
West (reference)	…	…	…	…	…	…
Midwest	1.31 (0.87-1.98)	.194	2.37 (1.29-4.35)	**.005**	0.78 (0.42-1.43)	.414
South	1.15 (0.78-1.68)	.485	1.82 (1.06-3.12)	**.029**	0.81 (0.44-1.48)	.499
Northeast	1.23 (0.81-1.88)	.332	1.45 (0.8-2.62)	.218	1.12 (0.58-2.15)	.736
Distance to study site^h^	0.99 (0.97-1.01)	.401	0.99 (0.96-1.02)	.406	0.97 (0.94-1.01)	.110
Insurance - private	1.41 (0.53-3.74)	.490	0.98 (0.3-3.24)	.971	0.58 (0.05-6.13)	.648
Insurance - Medicare	1.17 (0.87-1.57)	.313	0.97 (0.64-1.47)	.885	1.24 (0.77-2.01)	.373
Insurance - others	0.65 (0.43-0.97)	**.035**	0.61 (0.33-1.11)	.103	0.63 (0.35-1.17)	.144
Smoking history	0.8 (0.61-1.05)	.104	0.75 (0.51-1.1)	.146	0.81 (0.53-1.23)	.323
No. of other chronic conditions	0.59 (0.25-1.37)	.218	0.55 (0.16-1.92)	.349	0.7 (0.18-2.62)	.593
Other chronic conditions						
GERD	1.82 (0.72-4.6)	.204	1.92 (0.51-7.28)	.335	1.53 (0.35-6.63)	.570
CAD	1.81 (0.69-4.74)	.226	2.03 (0.52-7.99)	.312	1.3 (0.26-6.46)	.747
CHF	1.45 (0.48-4.45)	.511	1.57 (0.31-7.87)	.585	1.21 (0.22-6.74)	.825
COPD	1.24 (0.47-3.28)	.660	1.23 (0.31-4.88)	.767	1.19 (0.25-5.7)	.823
Diabetes	2.56 (1.01-6.44)	**.047**	2.49 (0.66-9.31)	.176	2.62 (0.58-11.79)	.209
PAH	2.53 (0.91-7.07)	.077	1.8 (0.41-7.95)	.438	4.18 (0.82-21.43)	.086
PVD^i^	…	…	…	…	…	…
Family history of ILD	1.16 (0.79-1.68)	.453	0.99 (0.62-1.57)	.955	1.51 (0.71-3.18)	.285
Biopsy	0.88 (0.4-1.9)	.739	0.69 (0.2-2.46)	.572	1.09 (0.38-3.15)	.872
Transbronchial biopsy	1.12 (0.51-2.46)	.787	1.26 (0.31-5.12)	.751	0.8 (0.28-2.28)	.672
SLBx	2.11 (0.98-4.56)	.057	3.92 (1.08-14.16)	**.037**	1.08 (0.38-3.08)	.882
FVC % predicted	1 (1-1.01)	.394	1 (0.99-1.02)	.445	1 (0.99-1.01)	.844
Dlco uncorrected % predicted	1 (0.99-1.01)	.575	1 (0.98-1.01)	.356	1 (0.98-1.01)	.904
Supplemental oxygen use	1.05 (0.78-1.41)	.757	1.18 (0.78-1.79)	.432	0.95 (0.59-1.54)	.832
Pulmonary rehabilitation	1.09 (0.73-1.65)	.664	1.2 (0.69-2.07)	.523	1.02 (0.52-1.99)	.951
ILD subtype						
IIP-IPF (reference)	…	…	…	…	…	…
IIP-non-IPF	0.36 (0.24-0.55)	**< .001**	…	…	…	…
Collagen vascular disease/autoimmune disease	1.32 (0.84-2.07)	.232	…	…	…	…
Hypersensitivity pneumonitis	0.63 (0.38-1.04)	.070	…	…	…	…
Other forms of ILD	0.82 (0.39-1.72)	.594	…	…	…	…
MDT discussion	0.94 (0.72-1.23)	.639	1.28 (0.87-1.87)	.206	0.56 (0.37-0.84)	**.006**
Antifibrotic medications (pirfenidone or nintedanib)	1.51 (1.09-2.07)	**.012**	1.51 (1.05-2.17)	**.028**	0.84 (0.31-2.26)	.729
Immunomodulatory medications	0.94 (0.67-1.33)	.734	0.55 (0.32-0.93)	**.027**	1.71 (1.07-2.75)	**.025**
Proton pump inhibitor	1.02 (0.72-1.45)	.899	1.08 (0.68-1.71)	.757	0.85 (0.46-1.57)	.614
Definite HRCT pattern	…	…	2.09 (1.33-3.3)	**.001**	…	…
Days from consent to diagnosis^j^	0.937 (0.892-0.984)	**.009**	0.939 (0.869-1.014)	.108	0.93 (0.868-0.996)	**.039**

*P* values < .05 are highlighted in boldface font. CAD = coronary artery disease; CHF = congestive heart failure; Dlco = carbon monoxide diffusing capacity; GERD = gastroesophageal reflux disease; HRCT = high-resolution CT; IIP = idiopathic interstitial pneumonia; ILD = interstitial lung disease; IPF = idiopathic pulmonary fibrosis; MDT = multidisciplinary team; PAH = pulmonary arterial hypertension; PVD = pulmonary vascular disease; SLBx = surgical lung biopsy.

a The multivariable logistic regression model analysis also included the following variables: sleep apnea, arrhythmia, cancer, DVT, embolism, obesity, Fatigue Severity Scale score, Leicester Cough Questionnaire score, Rand Short-Form 6-Dimension health-related quality of life score, and University of California, San Diego Shortness of Breath score. These variables are presented in e-Table 5.

b Deviance and Pearson goodness-of-fit statistics for multivariate logistic regression were 1,573.7 (*P* = .19) and 1,595.8 (*P* = .10), respectively.

c Deviance and Pearson goodness-of-fit statistics for multivariate logistic regression were 848.1 (*P* = .93) and 1,017.9 (*P* = .007), respectively.

d Deviance and Pearson goodness-of-fit statistics for multivariate logistic regression were 642.2 (*P* = .006) and 609.3 (*P* = .055), respectively.

e From multivariable logistic regression model with all variables excluding PVD as predictors. PVD was excluded from analysis due to small sample size. HRCT scan was excluded in the model for all participants, ILD type was excluded in the model for participants with IPF, and both HRCT scan and ILD type were excluded in the model for participants with non-IPF.

f Sample sizes for multivariable analyses were 1,576/1,992 observations for all participants, 957/1,227 observations for participants with IPF, and 601/765 observations for participants with non-IPF ILD.

g Expressed in units of 10 y.

h Expressed in units of 40.2 km.

i Excluded from the analysis due to small cells.

j Expressed in units of 365 d.

Adjusted logistic regression analyses also showed that among participants with IPF, antifibrotic use, SLBx, Midwest or South (compared with West) region, and definite HRCT pattern were significantly associated with higher diagnostic confidence, whereas Hispanic ethnicity and immunomodulatory medications were significantly associated with lower confidence. Furthermore, in participants with non-IPF ILD, immunomodulatory medications and longer time from consent to diagnosis were associated with higher confidence, whereas older age, male sex, and history of MDT discussion were associated with lower diagnostic confidence (Table 2).

The adjusted logistic models used in this analysis had acceptable discriminating ability, with area under the curve values differing significantly from 0.5 for all participants (0.69; 95% CI, 0.66-0.72; *P* < .0001), for participants with IPF (0.73; 95% CI, 0.69-0.77; *P* < .0001), and for participants with non-IPF (0.72; 95% CI, 0.68-0.76; *P* < .0001).

## Discussion

Given the high level of diagnostic discordance between expert centers, Ryerson et al^3^ proposed an ontological framework for classifying ILD, incorporating adherence to guideline criteria and clinical judgment to assign a level of diagnostic certainty. Real-world studies of ILD diagnostic confidence are important to understand how IPF/ILD diagnostic guidelines are used in clinical practice, with previous work describing the influence of IPF diagnostic likelihood on antifibrotic prescribing practices.^1^ However, before that, it is also important to understand patient and clinical factors that may be associated with higher or lower diagnostic confidence in real-world practice to raise awareness among physicians and ultimately improve the consistency of diagnosing patients with ILD.

Both adjusted and unadjusted analyses showed that clinicians were more confident in the diagnosis of IPF than non-IPF ILD, which may be related to factors such as better awareness, focused education, and the availability of firm imaging criteria.^2^^,^^10^ Notably, among participants with IPF, a history of SLBx and a definite UIP pattern on HRCT scan increased diagnostic confidence, consistent with diagnostic guidance.^2^^,^^10^

In both the adjusted and unadjusted analyses, physiological variables (eg, FVC, Dlco) and PROs were not associated with diagnostic confidence in either the IPF or the non-IPF population. However, both the adjusted and unadjusted analyses showed that the type of treatment at baseline entry to the registry was linked to diagnostic confidence, with immunomodulatory medications associated with lower confidence in participants with IPF but higher confidence in participants with non-IPF ILD, and antifibrotic medication use was associated with higher diagnostic confidence in participants with IPF only, suggesting a potential effect of diagnostic anchoring associated with treatments. Of note, participants in this cohort were recruited before antifibrotic medications were approved for non-IPF ILD in 2020^11^; therefore, this development is unlikely to have impacted diagnostic confidence in this analysis for that cohort. Furthermore, adjusted analysis showed that among all participants, diabetes was associated with higher diagnostic confidence, and a meta-analysis has further probed this potential association between IPF and diabetes.^12^ The higher diagnostic confidence of ILD in participants with diabetes is an intriguing finding that needs further investigation in other ILD cohorts, particularly given the potential for common pathophysiological pathways between diabetes and pulmonary fibrosis.^13^

Interestingly, the unadjusted analysis showed that living in rural areas was associated with higher diagnostic confidence in participants with IPF; however, it was not a significant factor in the adjusted analysis. Because our analysis was not designed to identify causality, we can only speculate on possible reasons why IPF could be diagnosed with more confidence in participants living in rural areas; for example, pulmonary practices in rural areas are busy clinics, without the option to offer MDT discussion, and practices may lack specialized resources and expertise. Clinical anchoring may be another possible reason for reinforcing an IPF diagnosis on entry to the registry. Additionally, exposure to specific environmental conditions may differ between rural and urban areas, leading to different presentations of ILD in different areas; however, our analysis was not able to investigate this further. Accordingly, additional studies are required to investigate this area further.

Another notable finding was that in the adjusted analysis, there was no statistically significant association between the strength of the diagnostic confidence and post-MDT diagnosis among study participants with IPF. In contrast, MDT discussion was associated with lower confidence in the post-MDT diagnosis among participants with a non-IPF diagnosis. Compared with our results, previous studies suggested that diagnosis of ILDs by an MDT is associated with higher diagnostic confidence and better interobserver agreement compared with individual clinicians, radiologists, and pathologists.^1^^,^^14^, ^15^, ^16^, ^17^ However, real-world use of MDTs may differ from studies evaluating the validity of the MDT approach through review of consecutive series of patients. Although IPF guidelines suggest that an MDT should discuss all suspected cases,^2^ there is general consensus that further discussion is unnecessary if a diagnosis is straightforward.^6^^,^^18^ It is possible, therefore, that our results reflect a bias in real-world practice, with MDTs discussing more low- than high-confidence cases. Additionally, where confidence is lacking due to a paucity of clear clinical, radiologic, or pathologic evidence, diagnostic confidence may not always be improved after MDT discussion.

In the adjusted analysis only, Hispanic ethnicity was associated with lower diagnostic confidence in all participants and in participants with IPF, which may be related to preconceptions that Hispanic populations may experience disproportionately higher exposure to environmental antigens and air pollutants^19^^,^^20^ and therefore may have a propensity toward developing hypersensitivity pneumonitis. A longer time since diagnosis was associated with higher diagnostic confidence in all participants and in participants with IPF and non-IPF ILD in the unadjusted analysis, but only in all participants and in participants with non-IPF ILD in the adjusted analysis, which is a result of the lower level of stringency of the unadjusted analysis and potentially reflects the differences in the natural course of IPF vs non-IPF ILD, the latter being associated with a slower disease course.^21^, ^22^, ^23^ Finally, in both the adjusted and unadjusted analyses, the association of older age and male sex with lower diagnostic confidence in participants with non-IPF ILD could reflect the relatively high median age and predominance of male participants in the IPF population (∼75%) compared with participants with non-IPF ILDs (< 50%).^7^ These findings indicate that cognitive biases may influence the confidence in which IPF is diagnosed; therefore, further investigations are required to eliminate such biases.

This analysis has some limitations, including its retrospective and observational nature. Additionally, assessments of diagnostic confidence did not necessarily follow strict ontological guidelines and potentially varied among investigators and geographic locations. It is also unknown how many practitioners in the PFF registry used thresholds from the diagnostic framework published by Ryerson et al.^3^ Participants may have been referred into the registry from a mixture of community or academic centers, and findings may have been affected by time since diagnosis in either scenario. However, because diagnostic confidence is assigned by academic PFF centers on review of the cases, this limitation may also be considered a strength, by providing a wider breadth of cases (community-academic and academic-academic).

Overall, it is hoped that these findings will help to improve awareness among practitioners that certain demographic and clinical factors may be consciously or unconsciously influencing their confidence in the diagnosis of IPF and non-IPF ILD. Tailored education may be useful for reducing such biases, particularly for patients of Hispanic ethnicity, and for improving consistency in diagnostic confidence, which may impact future care for patients with IPF and non-IPF ILD.

## Funding/Support

This analysis was funded by F. Hoffmann-La Roche, Ltd/Genentech, Inc. F. Hoffmann-La Roche, Ltd/Genentech, Inc. provide support for the Pulmonary Fibrosis Foundation.

## Financial/Nonfinancial Disclosures

The authors have reported to *CHEST* *Pulmonary* the following: M. B. S. has served as a consultant for Genentech, Boehringer Ingelheim, Bristol Myers Squibb, and United Therapeutics. S. G. is a full-time employee of Genentech, Inc. K. R. F. has received institutional funding from Boehringer Ingelheim and consulting fees from Arrowhead, AstraZeneca, Bellerophon, CSL Behring, Daewoong, DevPro, Dispersol, FibroGen, Genentech, Horizon, Immunet, Insilco, Lupin, NeRRe Therapeutics, Pliant, Polarean, Pure Health, PureTech, Respivant, Roche, Shionogi, Sun Pharmaceuticals, Trevi, United Therapeutics, and Vilcore. A. A. has served as a consultant for AbbVie, Boehringer Ingelheim, Inogen, Medscape, PatientMpower, and Roche; and has participated in an educational forum for Boehringer Ingelheim. None declared (R. V. I., Z. L.).
