# Supplementary material for: Demographic and Clinical Factors Associated With Diagnostic Confidence in Interstitial Lung Disease: Findings From the Pulmonary Fibrosis Foundation Patient Registry
Source: CHEST Pulm. 2024 Jul 15;2(4):100084. doi: 10.1016/j.chpulm.2024.100084 (PMC13418950; doi:10.1016/j.chpulm.2024.100084)
Supplement: Supplementary Material [file mmc1.pdf]

Supplementary material for

Demographic and Clinical Factors Associated with Diagnostic Confidence in Interstitial Lung Disease: Findings from the Pulmonary Fibrosis Foundation Patient Registry

Mary Beth Scholand, MD, Sachin Gupta, MD, Kevin R. Flaherty, MD, Rosalinda V. Ignacio, MS, Zhongze Li, MS, Ayodeji Adegunsoye, MD

Supplementary Material

e-TABLE 1 Types of Non-IPF ILDs[a] in Patients Enrolled in the Registry[1]

Non-IPF IIPs	CVD-ILDs	Hypersensitive pneumonitis
Fibrotic nonspecific interstitial pneumonia	Systemic sclerosis/scleroderma	Hot tub lung
Cellular nonspecific interstitial pneumonia	Polymyositis	Farmer's lung
Respiratory bronchiolitis-ILD	Dermatomyositis	Mold, down, and bird fancier's lung
Desquamative interstitial pneumonia	Antisynthetase syndrome	
Cryptogenic organizing pneumonia	Systemic lupus erythematous	
Acute interstitial pneumonia	Rheumatoid arthritis	
Lymphocytic interstitial pneumonia	Ankylosing spondylitis	
Pleuroparenchymal fibroelastosis	Mixed connective tissue disease	
IIP	Sjogren's syndrome	
Unclassifiable IIPs	Undifferentiated connective tissue disease	
	Antineutrophil cytoplasmic antibody-associated vasculitis	
	Pulmonary capillaritis	
	Granulomatosis with polyangiitis	

CVD = collagen vascular disease; IIP = idiopathic interstitial pneumonia; ILD = interstitial lung disease; IPF = idiopathic pulmonary fibrosis.

[a]The patterns of CT scans of participants with non-IPF conditions were not recorded in the Registry.

e-TABLE 2 Baseline Demographic and Clinical Characteristics at Enrollment by Diagnosis Confidence Level (High vs Medium/Low): Participants with IPF

**Characteristics**	**High Confidence (n = 977)**	**Medium / Low Confidence (n = 250)**
Age, mean (SD) [median], y	70.7 (7.8) [71.0]	69.9 (8.8) [70.0]
Male	741 (75.8)	186 (74.4)
**Race[a]***		
White	919 (94.1)	228 (91.2)
Black or African American	12 (1.2)	4 (1.6)
Other	18 (1.8)	13 (5.2)
Hispanic	45 (4.7)	15 (6.2)
**Rural***	127 (13.0)	21 (8.4)
Region		
West	148 (15.1)	47 (18.8)
Midwest	214 (21.9)	39 (15.6)
South	409 (41.9)	104 (41.6)
Northeast	206 (21.1)	60 (24.0)
Insurance - private	961 (98.4)	243 (97.2)
Insurance - Medicare	672 (68.8)	157 (62.8)
**Insurance - others***	89 (9.1)	34 (13.6)
Smoking history	612 (62.6)	171 (68.4)
No. of other chronic conditions, mean (SD) [median]	1.6 (1.3) [1.0]	1.7 (1.5) [1.0]
Other chronic conditions		
GERD	170 (17.4)	37 (14.8)
CAD	249 (25.5)	64 (25.6)
CHF	34 (3.5)	11 (4.4)
COPD	81 (8.3)	29 (11.6)
Diabetes	204 (20.9)	47 (18.8)
PAH	60 (6.1)	14 (5.6)
**PVD***	15 (1.5)	9 (3.6)
Family history of ILD	176 (18.0)	42 (16.8)
**Biopsy*****	335 (34.3)	58 (23.2)
Transbronchial biopsy	42 (4.3)	12 (4.8)
**SLBx*****	296 (30.3)	40 (16.0)
FVC % predicted, mean (SD) [median], %	68.2 (16.8) [67.5]	68.1 (18.7) [65.8]
FVC % predicted, categorical		
< 50%	130 (13.3)	39 (15.6)
50% to < 80%	573 (58.6)	132 (52.8)
≥80%	234 (24.0)	63 (25.2)
DLco uncorrected % predicted, mean (SD) [median], %	40.7 (17.7) [38.9]	41.9 (16.8) [39.0]
DLco uncorrected % predicted, categorical		
< 50%	689 (70.5)	163 (65.2)
50% to < %80%	188 (19.2)	50 (20.0)

Characteristics	High Confidence (n = 977)	Medium / Low Confidence (n = 250)
≥ 80%	17 (1.7)	6 (2.4)
Supplemental oxygen use	474 (48.5)	116 (46.4)
**MDT discussion***	430 (44.4)	89 (35.6)
**Antifibrotic medications (pirfenidone or nintedanib)*****	638 (65.3)	128 (51.2)
**Immunomodulatory medications***	99 (10.1)	38 (15.2)
Proton pump inhibitor	190 (19.4)	48 (19.2)
**Definite HRCT pattern****	201 (38.2)	35 (25.7)
**Days from consent to diagnosis, mean (SD) [median]***	-945.8 (968.4) [-660.5]	-773.6 (946.9) [-477.5]

Data are n (%) unless indicated otherwise. Categories with *P*-values < .05 are highlighted in bold.

*P < .05, **P < .01, ***P < .001.

CAD = coronary artery disease; CHF = congestive heart failure; COPD = chronic obstructive pulmonary disease; DLco = carbon monoxide diffusing capacity; DVT = deep vein thrombosis; FVC = forced vital capacity; GERD = gastroesophageal reflux disease; HRCT = high-resolution computed tomography; ILD = interstitial lung disease; IPF = idiopathic pulmonary fibrosis; MDT = multidisciplinary team; PAH = pulmonary arterial hypertension; PVD = pulmonary vascular disease; QOL = quality of life; SD = standard deviation; SF-6D = Short-Form 6-Dimension; SLBx = surgical lung biopsy; UCSD = University of California, San Diego.

[a]Excludes 33 participants with race not reported or unknown race.

e-TABLE 3 Baseline Demographic and Clinical Characteristics at Enrollment by Diagnosis Confidence Level (High vs Medium/Low): Participants with Non-IPF ILD

**Characteristics**	**High Confidence (n = 543)**	**Medium / Low Confidence (n = 222)**
**Age, mean (SD) [median], y****	62.3 (12.2) [64.0]	65.3 (10.1) [67.0]
**Male****	213 (39.2)	111 (50.0)
**Race[a]***		
White	435 (80.1)	194 (87.4)
Black or African American	76 (14.0)	14 (6.3)
Other	17 (3.1)	7 (3.2)
Hispanic	41 (7.9)	23 (10.8)
Rural	63 (11.6)	33 (14.9)
Region		
West	96 (17.7)	42 (18.9)
Midwest	139 (25.6)	62 (27.9)
South	170 (31.3)	78 (35.1)
Northeast	138 (25.4)	40 (18.0)
Insurance - private	538 (99.1)	220 (99.1)
Insurance - Medicare	257 (47.3)	111 (50.0)
Insurance - others	70 (12.9)	32 (14.4)
Smoking history	265 (48.8)	114 (51.4)
No. of other chronic conditions, mean (SD), median	1.4 (1.5) [1.0]	1.3 (1.3) [1.0]
Other chronic conditions		
GERD	83 (15.3)	27 (12.2)
CAD	69 (12.7)	36 (16.2)
CHF	28 (5.2)	11 (5.0)
COPD	41 (7.6)	26 (11.7)
Diabetes	97 (17.9)	30 (13.5)
**PAH****	67 (12.3)	12 (5.4)
PVD	8 (1.5)	6 (2.7)
Family history of ILD	50 (9.2)	15 (6.8)
Biopsy	248 (45.7)	87 (39.2)
Transbronchial biopsy	51 (9.4)	23 (10.4)
**SLBx***	200 (36.8)	61 (27.5)
FVC % predicted, mean (SD) [median], %	67.6 (19.5) [66.6]	67.6 (20.5) [66.7]
FVC % predicted, categorical		
< 50%	94 (17.3)	48 (21.6)
50% to < 80%	309 (56.9)	106 (47.7)
≥ 80%	124 (22.8)	58 (26.1)
DLco uncorrected % predicted, mean (SD) [median], %	44.5 (17.5) [42.7]	45.3 (18.4) [42.3]
DLco uncorrected % predicted, categorical		
< 50%	335 (61.7)	129 (58.1)
50% to < %80%	152 (28.0)	63 (28.4)
≥ 80%	24 (4.4)	12 (5.4)

**Characteristics**	**High Confidence (n = 543)**	**Medium / Low Confidence (n = 222)**
Supplemental oxygen use	223 (41.1)	89 (40.1)
**MDT discussion****	197 (36.3)	108 (48.6)
Antifibrotic medications (pirfenidone or nintedanib)	19 (3.5)	9 (4.1)
**Immunomodulatory medications****	180 (33.1)	49 (22.1)
Proton pump inhibitor	71 (13.1)	30 (13.5)
**Days from consent to diagnosis, mean (SD) [median]*****	-1,277.3 (1,368.0) [-824.5]	-901.6 (1,022.7) [-563.0]

Data are n (%) unless indicated otherwise. Categories with *P*-values < .05 are highlighted in bold.

*P < .05, **P < .01, ***P < .001.

CAD = coronary artery disease; CHF = congestive heart failure; COPD = chronic obstructive pulmonary disease; DLco = carbon monoxide diffusing capacity; DVT = deep vein thrombosis; FVC = forced vital capacity; GERD = gastroesophageal reflux disease; ILD = interstitial lung disease; IPF = idiopathic pulmonary fibrosis; MDT = multidisciplinary team; PAH = pulmonary arterial hypertension; PVD = pulmonary vascular disease; QOL = quality of life; SD = standard deviation; SF-6D = Short-Form 6-Dimension; SLBx = surgical lung biopsy; UCSD = University of California, San Diego.

[a]Excludes 22 participants with race not reported or unknown race.

e-TABLE 4 Unadjusted Logistic Regression Analyses for Diagnosis Confidence Level (High vs Medium/Low)

	**All Participants**		**Participants with IPF**		**Participants with Non-IPF ILD**	
**Variables**	**Unadjusted OR (95% CI)[a,b]**	***P* Value**	**Unadjusted OR (95% CI)[a,b]**	***P* Value**	**Unadjusted OR (95% CI)[a,b]**	***P* Value**
Age[c]	0.99 (0.9-1.1)	.860	1.12 (0.94-1.33)	.193	0.79 (0.69-0.91)	**.001**
Male	0.99 (0.8-1.23)	.950	1.08 (0.78-1.49)	.635	0.65 (0.47-0.88)	**.006**
Race						
White (reference)						
Black or African American	1.52 (0.91-2.56)	.111	0.74 (0.24-2.33)	.612	2.42 (1.34-4.39)	**.004**
Other	0.55 (0.31-0.95)	**.034**	0.34 (0.17-0.71)	**.004**	1.08 (0.44-2.65)	.861
Hispanic	0.68 (0.46-1.01)	.058	0.75 (0.41-1.38)	.358	0.7 (0.41-1.2)	.200
BMI	0.99 (0.97-1)	.109	0.99 (0.96-1.02)	.408	0.99 (0.96-1.01)	.305
Rural	1.11 (0.8-1.53)	.540	1.63 (1-2.64)	**.048**	0.75 (0.48-1.18)	.217
Region						
West (reference)						
Midwest	1.27 (0.92-1.77)	.147	1.74 (1.09-2.8)	**.022**	0.98 (0.61-1.57)	.936
South	1.16 (0.86-1.56)	.322	1.25 (0.84-1.85)	.267	0.95 (0.61-1.5)	.836
Northeast	1.25 (0.9-1.74)	.177	1.09 (0.7-1.69)	.698	1.51 (0.91-2.5)	.110
Distance to study site[d]	1 (0.98-1.01)	.600	1 (0.98-1.03)	.718	0.98 (0.96-1.01)	.179
Insurance - private	1.39 (0.63-3.05)	.415	1.73 (0.7-4.25)	.232	0.98 (0.19-5.08)	.979
Insurance - Medicare	1.2 (0.97-1.48)	.093	1.31 (0.98-1.74)	.072	0.9 (0.66-1.23)	.502
Insurance - others	0.72 (0.53-0.98)	**.035**	0.64 (0.42-0.97)	**.036**	0.88 (0.56-1.38)	.574
Smoking history	0.89 (0.72-1.1)	.302	0.77 (0.58-1.04)	.091	0.9 (0.66-1.23)	.522
No. of other chronic conditions	0.98 (0.91-1.06)	.675	0.92 (0.83-1.01)	.089	1.04 (0.93-1.16)	.463
Other chronic conditions						
GERD	1.27 (0.95-1.71)	.110	1.21 (0.82-1.78)	.328	1.3 (0.82-2.08)	.265
Sleep apnea	1.06 (0.84-1.35)	.612	0.96 (0.7-1.31)	.796	1.19 (0.82-1.73)	.368
Arrhythmia	1.1 (0.77-1.57)	.598	1.07 (0.68-1.7)	.767	1.07 (0.61-1.89)	.805
CAD	0.98 (0.76-1.27)	.901	0.99 (0.72-1.37)	.971	0.75 (0.49-1.16)	.202

CHF	0.87 (0.53-1.43)	.583	0.78 (0.39-1.57)	.491	1.04 (0.51-2.13)	.909
COPD	0.66 (0.47-0.93)	**.016**	0.69 (0.44-1.08)	.104	0.62 (0.37-1.03)	.067
Cancer	0.9 (0.68-1.18)	.428	0.78 (0.55-1.1)	.158	1.02 (0.65-1.6)	.938
DVT	0.87 (0.48-1.58)	.637	0.56 (0.28-1.13)	.106	2.07 (0.59-7.24)	.253
Diabetes	1.27 (0.96-1.67)	.092	1.14 (0.8-1.62)	.467	1.39 (0.89-2.17)	.144
Embolism	0.88 (0.48-1.64)	.696	0.52 (0.25-1.09)	.084	2.36 (0.68-8.13)	.174
Obesity	0.67 (0.52-0.87)	**.003**	0.62 (0.44-0.88)	**.007**	0.76 (0.51-1.12)	.165
PAH	1.56 (1.01-2.41)	**.044**	1.1 (0.61-2.01)	.748	2.46 (1.3-4.65)	**.005**
PVD	0.47 (0.24-0.9)	**.024**	0.42 (0.18-0.97)	**.041**	0.54 (0.18-1.57)	.257
Family history of ILD	1.27 (0.93-1.74)	.130	1.09 (0.75-1.57)	.654	1.4 (0.77-2.55)	.272
Biopsy	1.4 (1.12-1.75)	**.003**	1.73 (1.25-2.38)	**< .001**	1.3 (0.95-1.79)	.101
Transbronchial biopsy	0.81 (0.54-1.22)	.316	0.89 (0.46-1.72)	.730	0.9 (0.53-1.51)	.681
SLBx	1.78 (1.39-2.27)	**< .001**	2.28 (1.58-3.29)	**< .001**	1.54 (1.09-2.17)	**.014**
FVC % predicted	1 (0.99-1.01)	.867	1 (0.99-1.01)	.907	1 (0.99-1.01)	.985
DLco uncorrected % predicted	1 (0.99-1)	.135	1 (0.99-1)	.354	1 (0.99-1.01)	.592
Supplemental oxygen use	1.1 (0.9-1.36)	.356	1.09 (0.82-1.44)	.550	1.04 (0.76-1.43)	.803
Pulmonary rehabilitation	1.09 (0.79-1.51)	.599	1 (0.65-1.53)	.991	1.21 (0.73-2.03)	.456
Fatigue Severity Scale score	1.03 (0.97-1.09)	.324	1.02 (0.94-1.1)	.708	1.06 (0.97-1.16)	.178
Leicester Cough Questionnaire score	1 (0.98-1.03)	.799	1 (0.96-1.04)	.995	1 (0.96-1.04)	.914
Rand SF-6D Health-related QOL score	1.47 (0.57-3.78)	.428	2.16 (0.58-8.03)	.250	0.5 (0.12-2.04)	.336
UCSD Shortness of Breath score	1 (1-1)	.876	1 (0.99-1)	.625	1 (1-1.01)	.200
ILD subtype						
IIP - IPF (reference)						
IIP - non-IPF	0.33 (0.25-0.45)	**< .001**	--	--	--	--
Collagen vascular disease / autoimmune diseases	1.12 (0.82-1.52)	.478	--	--	--	--
Hypersensitivity pneumonitis	0.58 (0.4-0.83)	**.003**	--	--	--	--
Other forms of ILD	0.6 (0.34-1.06)	.076	--	--	--	--
MDT discussion	0.98 (0.79-1.21)	.851	1.42 (1.07-1.9)	**.017**	0.6 (0.44-0.82)	**.002**
Antifibrotic medications (pirfenidone or nintedanib)	1.86 (1.49-2.33)	**< .001**	1.79 (1.35-2.38)	**< .001**	0.86 (0.38-1.93)	.711

Immunomodulatory medications	0.99 (0.76-1.3)	.970	0.63 (0.42-0.94)	**.024**	1.75 (1.22-2.52)	**.003**
Proton pump inhibitor	1.05 (0.79-1.38)	.746	1.02 (0.71-1.45)	.930	0.96 (0.61-1.52)	.870
Definite HRCT pattern	--	--	2.12 (1.51-2.98)	**< .001**	--	--
Days from consent to diagnosis[e]	0.925 (0.889-0.962)	**< .001**	0.927 (0.874-0.984)	**.012**	0.907 (0.86-0.956)	**< .001**

P-values < .05 are highlighted in bold.

BMI = body mass index; CAD = coronary artery disease; CHF = congestive heart failure; CI = confidence interval; COPD = chronic obstructive pulmonary disease; DLco = carbon monoxide diffusing capacity; DVT = deep vein thrombosis; FVC = forced vital capacity; GERD = gastroesophageal reflux disease; HRCT = high-resolution computed tomography; IIP = idiopathic interstitial pneumonia; ILD = interstitial lung disease; IPF = idiopathic pulmonary fibrosis; MDT = multidisciplinary team; OR = odds ratio; PAH = pulmonary arterial hypertension; PVD = pulmonary vascular disease; QOL = quality of life; SF-6D = Short-Form 6-Dimension; SLBx = surgical lung biopsy; UCSD = University of California, San Diego.

[a]From separate simple logistic regression models with each variable as predictor.

[b]Sample sizes for multivariable analyses were 1,576/1,992 observations for all participants, 957/1,227 observations for participants with IPF, and 601/765 observations for participants with non-IPF ILD.

[c]Expressed in units of 10 years.

[d]Expressed in units of 40.2 km.

[e]Expressed in units of 365 days.

e-TABLE 5 Additional Variables Included in the Adjusted Logistic Regression Analyses for Diagnosis Confidence Level (High vs Medium/Low)

	All Participants		Participants with IPF		Participants with Non-IPF ILD	
**Variables[a]**	**Adjusted OR (95% CI)[b,c]**	***P* Value**	**Adjusted OR (95% CI)[b,c]**	***P* Value**	**Adjusted OR (95% CI)[b,c]**	***P* Value**
Other chronic conditions						
Sleep apnea	1.58 (0.63-3.94)	.325	1.23 (0.32-4.64)	.763	2.02 (0.48-8.49)	.335
Arrhythmia	1.81 (0.72-4.59)	.209	1.77 (0.46-6.85)	.406	1.38 (0.32-5.9)	.666
Cancer	1.55 (0.62-3.9)	.349	1.6 (0.42-6.12)	.495	1.68 (0.39-7.23)	.484
DVT	1.47 (0.46-4.66)	.512	1.07 (0.21-5.3)	.937	2.45 (0.31-19.03)	.392
Embolism	1.11 (0.35-3.5)	.861	0.62 (0.13-3.08)	.562	3.82 (0.48-30.42)	.205
Obesity	0.94 (0.38-2.36)	.903	0.81 (0.21-3.09)	.755	0.89 (0.21-3.77)	.880
Fatigue Severity Scale score	1.05 (0.95-1.16)	.322	1.09 (0.95-1.27)	.226	1.07 (0.92-1.25)	.396
Leicester Cough Questionnaire score	0.99 (0.95-1.03)	.738	0.99 (0.94-1.05)	.809	1.02 (0.96-1.08)	.496
Rand SF-6D Health-Related QOL score	3 (0.62-14.53)	.173	3.31 (0.35-31.23)	.295	2 (0.18-22.28)	.572
UCSD Shortness of Breath score	1 (0.99-1.01)	.602	1 (0.99-1.01)	.946	1 (0.99-1.02)	.687

CI = confidence interval; DVT = deep vein thrombosis; ILD = interstitial lung disease; IPF = idiopathic pulmonary fibrosis; OR = odds ratio; QOL = quality of life; SF-6D = Short-Form 6-Dimension; UCSD = University of California, San Diego.

[a]The multivariable logistic regression model analysis also included all factors presented in Table 2 of the main manuscript.

[b]From multivariable logistic regression model with all variables excluding PVD as predictors. PVD was excluded from analysis due to small sample size. HRCT was excluded in the model for all participants, ILD type was excluded in the model for participants with IPF, and both HRCT and ILD type were excluded in the model for participants with non-IPF.

[c]Sample sizes for multivariable analyses were 1,576/1,992 observations for all participants, 957/1,227 observations for participants with IPF, and 601/765 observations for participants with non-IPF ILD.
